# Leaving Home for Marital and Non-marital Reasons in the Netherlands, 1850–1940: The Impact of Parental Death and Parental Remarriage

**DOI:** 10.1007/s10680-022-09614-7

**Published:** 2022-03-30

**Authors:** Matthias Rosenbaum-Feldbrügge

**Affiliations:** 1 Federal Institute for Population Research, Friedrich-Ebert-Allee 4, 65185 Wiesbaden, Germany; 2 Radboud Group for Historical Demography and Family History, Radboud University Nijmegen, Nijmegen, The Netherlands

**Keywords:** Leaving home, Life-course, Historical demography, Parental death, Competing risk

## Abstract

Previous research shows that parentally bereaved children in north-western Europe in the past left home earlier than children who lived together with both biological parents. This article analyses the mechanisms behind this phenomenon with a special focus on the routes out of the parental household and the entry of step-parents and step-siblings. The Historical Sample of the Netherlands is exploited which contains detailed information about household composition and life courses of more than 22,000 female and male adolescent and young adult children born between 1850 and 1922. Event-history analysis is applied, and two exclusive routes out of the parental household, for marital and non-marital reasons, are studied in a competing risk design. The results show that parental loss does not increase the risk of early marriage before age 23, but strongly enhances the chances for leaving home for non-marital reasons, which are mainly work-related. This is especially true in case of maternal loss. No support is found for the hypothesis that the entry of a step-parent and step-siblings increases the risk of leaving home compared to living with a single widowed parent. Tensions with step-parents therefore do not suffice to explain why parentally bereaved children left earlier for non-marital reasons. Instead, we argue that children’s exit was in the interest of both the single widowed parent and the bereaved child.

## Introduction

In north-western Europe in the nineteenth and the first decades of the twentieth century, children often moved out of the parental home at young ages to work as domestic servants or farmhands with other families, a phenomenon which is referred to as life-cycle service (Hajnal, 1983; Laslett, 1977; van Poppel et al., 2004). Those who stayed at the parental home during adolescence and early adulthood typically left upon marriage to establish an own independent household with their partner. In contrast to today’s western societies, where leaving the parental household is typically associated with individual autonomy and higher education, leaving home in the past was therefore mainly related to either employment or family formation.

An important determinant of the age at leaving home in both historical and contemporary societies is the family structure in which the child grows up. Studies on western Europe in the nineteenth and beginning of the twentieth century found conclusive evidence that parental death was associated with earlier exit out of the parental home (Alter & Capron, 2004; Bras & Kok, 2004; Bras & Neven, 2007; Dribe, 2003; Lundh & Öberg, 2018), but the mechanisms behind this phenomenon remain unclear. Historical demographers have argued that the loss of a parent resulted in earlier home leaving because of high remarriage rates and tensions with step-parents that pushed the children out of the parental household (Bras & Kok, 2004; Dribe, 2003), a phenomenon which is often found in studies on contemporary western populations (Aquilino, 1991; Blaauboer & Mulder, 2010; Goldscheider & Goldscheider, 1998; van den Berg et al., 2018). To our knowledge, however, the role of step-parents on leaving home in the past has never been studied empirically (Bras & Kok, 2004; Dribe, 2003). This article therefore contributes to the existing literature by accounting for the arrival of step-fathers, step-mothers, and step-siblings in the household. This allows us to examine if the high risk of home leaving associated with parental death might be attributed to tensions in the family home triggered by the entry of a step-parent or to parental death and single parenthood per se. Furthermore, we will control for the child’s age at parental loss and at the entry of the step-parent to analyse if the timing of these events mattered with regard to home leaving.

As a second major contribution, we study two mutually exclusive pathways out of the parental home: Leaving home with a partner to live in a marital union and leaving home without a partner, mostly for work-related reasons. Research on contemporary populations shows that the determinants of leaving home differ considerably by the specific route chosen (Bernhardt et al., 2005; Blaauboer & Mulder, 2010; Cherlin et al., 1995; De Jong Gierveld et al., 1991; Dribe & Stanfors, 2005; Goldscheider & Goldscheider, 1998; Holdsworth, 2000). Nevertheless, previous research on home leaving in historical western and northern European populations grouped all available routes together or considered only one specific route (for a recent exception see Lundh & Öberg, 2018 on the Swedish city Gothenburg). As we expect that the determinants of the leaving home process vary considerably between marital and non-marital exit, we will study both pathways within a competing-risk framework, which allows us to regard the routes as alternative options.

Finally, it has recently been criticized that determinants of leaving home in historical populations have only been studied by using small regional samples (Day, 2018). This article will therefore contribute to the existing literature on leaving home in the past by being the first one that exploits a nationally representative sample. We use the Historical Sample of the Netherlands (HSN) which contains detailed information on the life courses of roughly 37,000 individuals born in the whole of the Netherlands between 1850 and 1922.

## Historical Context and Theoretical Background

### Life-Cycle Service and Leaving Home in the Past

Early home leaving in contemporary Western society is considered problematic because it generally interferes with obtaining educational attainment (McLanahan & Percheski, 2008). In north-western Europe in the nineteenth century, in contrast, it was common practice that sons and daughters left the nuclear family home at a relatively early age to live and work in another family as domestic servant, agricultural worker, or apprentice (Dribe, 2000; Mönkediek et al., 2016; van Poppel et al., 2004). Youth migration rates in the Netherlands in the period of consideration began to increase slowly from the age of 12 onwards, when the majority of children finished school (Kok, 1997). On average, youth migrants left the parental home for the first time at the age of 19 and the age at leaving home remained more or less stable during the nineteenth century (Kok, 1997). Working-age children were usually hired by their employers on a yearly basis (Dribe, 2000; Paping, 1999) and children working in service often returned to their parental home between employments (Kok, 1997). Young adults typically worked in service until they had accumulated enough savings to contract a marriage (de Moor & van Zanden, 2010; Dribe et al., 2014). Dutch children who did not leave home during childhood and young adulthood typically exited the parental household upon marriage and founded an independent nuclear household with their partner. Life-cycle service and the social norm to establish an own household upon marriage were important explanatory factors for the western European marriage pattern which is characterized by comparatively high ages at first marriage and high proportions of never-marrying individuals (Dribe et al., 2014; Hajnal, 1965, 1982).

Not only the leaving home behaviour, but also the social norms concerning the resource flow between generations have changed remarkably in north-western Europe over time. Whereas offspring in the contemporary western world is often supported financially by their parents up until adulthood, children in the period of consideration were expected to contribute to the family economy from an early age onwards (de Regt, 2004). Parents were also entitled to the entire earnings of their living-in children and generally made full use of this right (Bras & Kok, 2004). After having left the parental home, particularly children from the lower social classes still had to hand over their wage as children’s income was important to working class families to obtain a reasonable standard of living. Only after entry into marriage, children were not expected to contribute to the parents’ household budget any longer, and as a consequence the parental household lost the newly wed child as a productive asset (Bras & Kok, 2004).

Consequently, the decision to enter life-cycle service was directed by mutual obligations and dependencies between parents and children, and subject to a bargaining process in which costs and benefits both for the parental household and the child were taken into consideration (Dribe, 2000). The final decision about entrance into life-cycle service for minor children below the age of 23 (below the age of 21 after 1905), however, was typically made by the parents (Bras & Kok, 2004). The power relations between parents and children are also symbolized by the fact that parents were entitled to object to their children’s partner choice (Vulsma, 1988): Up until the age of 30, children who intended to get married needed their parent’s consent and for children below the age of 23 parental objection could not even be overruled by the court.

Earlier research on the Netherlands indicates that the determinants of leaving home differed substantially between sons and daughters (Bras & Kok, 2004). For instance, sons of elite members reported very high risks of leaving the parental home, whereas their sisters did not, which might be related to elite boys’ higher chance to go to boarding schools. In addition, girls’ leaving home behaviour was generally more affected by the presence of older and younger siblings than boys’, and particularly the presence of many younger sisters pushed girls out of the parental household. Due to these gender differences, in the empirical analysis we run regressions for sons and daughters separately.

### The Impact of Parental Death on Leaving Home Behaviour

Whereas parental divorce was a very exceptional event in the Netherlands in the second half of the nineteenth and the beginning of the twentieth century (Kalmijn, 2008), many children were confronted with parental death. Nearly one out of four children born between 1850 and 1879 had lost a mother and/or father by the age of 15. Even though this share decreased over time due to lower adult mortality risk, more than 13% of children born in the period 1900–1922 had lost a parent before the age of 15 (van Poppel et al., 2013). After the death of a parent, a co-guardian was appointed to half-orphaned children under the age of 23 (after 1905 the age of 21), who functioned as a supervisor of the main guardian (typically the surviving parent), but was usually not engaged in the upbringing of the minor children.

Earlier studies on historical Western populations unambiguously found that parental death was associated with children’s earlier exit out of the parental home. In the Dutch coastal province of Zeeland, Bras (2003, 2004) estimated that both maternal and paternal death increased daughter’s risk of entering service outside the parental home and the risk of migrating across provincial borders. Similar effects were found among Zeeland boys and children in the central province of Utrecht (Bras & Kok, 2004; Kok, 1997). Also children born in Eastern Belgium (Alter & Capron, 2004; Bras & Neven, 2007), rural Southern Sweden (Dribe, 2000, 2003), and in the USA (Galenson, 1987; Steckel, 1996) in the nineteenth century revealed higher risks of leaving home following the death of a parent.

Research on individuals born in the second half of the twentieth century has shown that the family structure has different effects on the available routes out of the parental home (Bernhardt et al., 2005; Blaauboer & Mulder, 2010; Cherlin et al., 1995; De Jong Gierveld et al., 1991; Dribe & Stanfors, 2005; Goldscheider & Goldscheider, 1998; Holdsworth, 2000). In their recent study on the leaving home process in Gothenburg in the first half of the twentieth century, Lundh and Öberg (2018) showed that this also applied to historical populations. Their results suggest that living with a widowed parent increased daughter’s chances to leave for non-marital reasons but not for entering a marriage. In studies on the impact of family structure on leaving home in historical populations, it is therefore important to differentiate between leaving home for marital and non-marital reasons.

In line with earlier research on the Netherlands, we assume that parental death increased the chances for leaving the parental household for non-marital reasons (Bras & Kok, 2004). The loss of a parent might have reduced the claims and the control of the parental family and generated possibilities to leave the household (Bras, 2003). Furthermore, parental death might be associated with severe consequences such as decreasing living standards (Oris & Ochiai, 2002) and a reduction in parental quality and quantity (Bernhardt et al., 2005) that push the child out of the parental home. As a consequence, bereaved children had to find work more rapidly than was anticipated in order to provide for themselves and also for their family (Bras & Kok, 2004). If work was not available in the community itself, children were sent away to find an occupation elsewhere.

On the contrary, we expect that parental death did not systematically affect leaving home for union formation. Marriage in the Netherlands in the nineteenth and beginning of the twentieth century was highly determined by financial, religious, and social constraints (Rosenbaum-Feldbrügge & Debiasi, 2019; Suanet & Bras, 2010). As parental death in childhood introduced financial instabilities (Oris & Ochiai, 2002), adult children might have delayed marriage until a certain standard of living was regained that facilitated entry into marriage. Parental death is therefore expected not to be associated with a rush into marriage. Moreover, widowed parents themselves did not have an interest in their children’s early marriage because married children usually did not contribute to the household income any longer (de Regt, 2004). As a result, widowed parents who were dependent on their adult children’s earnings might have objected to their children’s desire for marriage. Accordingly, we formulate the following hypotheses:

#### 1a

Parental death is associated with earlier home leaving for non-marital reasons.

#### 1b

Parental death is not associated with earlier home leaving for marital reasons.

Even though a clear link between parental death and early leaving of the parental household in historical populations has been established in previous work, the mechanisms behind this relationship remain unclear. Bras and Kok (2004) as well as Dribe (2003) suggested that half-orphaned children left the parental household earlier because of potential tensions with step-parents and step-siblings, but in their studies the authors did not control for their arrival. Remarriage occurred frequently in the Netherlands and especially widowers with young children had a strong interest in finding a new partner because they needed support with childcare services and domestic work. Accordingly, it has been found that remarriage rates were high for young widowers (Rosenbaum-Feldbrügge, 2018). For widows, however, and particularly for those with young children, remarriage rates were much lower (van Poppel, 1995).

In line with earlier research on contemporary Western populations, we assume that the entry of a step-parent and step-siblings increased the risk of leaving the parental home in childhood, adolescence, and young adulthood as parental remarriage has been associated with tensions in the family (Aquilino, 1991; Blaauboer & Mulder, 2010; Goldscheider & Goldscheider, 1998; van den Berg et al., 2018). Therefore, parental remarriage as well as the entry of step-siblings are expected to increase the risk of home leaving even compared to living with a single widowed parent. Furthermore, we assume that the timing of the entry of the step-parent mattered. Step-parents who had entered before the research person turned 12 years are expected to have a less disruptive impact than step-parents that entered after age 12. These considerations lead to the article’s final three hypotheses:

#### 2a

The entry of a step-parent both after and before the age of 12 accelerates the leaving home process compared to one-parent families.

#### 2b

The presence of step-siblings is generally associated with a faster transition to first leaving home.

#### 2c

The entry of a step-parent after age 12 is associated with a faster transition to first leaving home than the entry of a step-parent before age 12.

## Data, Methods and Variables

### Data and Sample Selection

The 2010.01 release of the Historical Sample of the Netherlands (HSN) follows the life courses of a representative sample of more than 37,000 male and female individuals, so-called research persons, who were born in the period 1850–1922. The HSN is based on birth, marriage and death certificates as well as on population registers which were introduced in the whole of the country in 1850. Population registers are a unique data source as they contain continuously updated information about demographic events of all the household members, such as birth, migration, and death (Mandemakers, 2002). This enables us to identify changes in the research persons’ household composition, for example the death of a parent, the entry of a step-parent, and most importantly the age at leaving home. In addition, information about occupation and religion is available in the registers, which are considered important predictor variables. The sampling strategy ensured that research persons are not clustered within households, which implies that each research person has a unique set of parents. Moreover, research persons who migrated within the country are followed and not lost from observation. From the end of the 1930s onwards, population registers were replaced by personal cards that do not contain information about the household structure. Therefore, the period of observation ends in 1940.

Research persons need to satisfy the following four criteria to be included in the analysis. First, they have to survive and have to be observed until the starting age of 14 years old. Especially due to high infant and child mortality risk, this reduces the number of research persons considerably from 37,173 to 25,952 individuals. Second, household information must be available in order to identify changes in the household composition. Third, research persons whose parents could not be identified without ambiguity, such as illegitimate children, and who were not living in the parental home at the age of 12 were excluded. Moreover, we decided to remove research persons that were abandoned by one of their parents for unknown reasons. This results in a sample size of 21,486. Finally, 43 individuals are removed because we do not have any information about their father’s socioeconomic status. The final sample therefore includes 10,655 female and 10,788 male research persons, which amounts to a total of 21,443 research persons whose leaving home behaviour is studied in this article. Individuals that leave the parental household and move to a household where no partner is present are defined as non-marital movers. Individuals are defined as marital movers when they leave the parental household and move into a household where a partner is present. Children who get married but do not leave the parental household are therefore not regarded as home leavers as long as they stay in the parental home.

### Methods

Event-history analysis (Cox proportional hazard models) is employed to examine the effect of family structure on first leaving the parental home. Event-history analysis calculates the expected duration of time until a certain event like leaving home occurs given a set of control variables such as the family structure or socioeconomic status. In the regression tables hazard ratios are presented. A coefficient above 1.00 is associated with a higher chance of home leaving, whereas a coefficient below 1.00 is associated with a reduced chance of home leaving. Higher chance of home leaving actually means that the event occurs earlier and/or more often compared to a reference group. Hence, in the regression models not only the timing but also the propensity of home leaving is studied.

The observation period starts with the 14th birthday of the research person and ends with the first occurrence of one of the following events: the event of interest (first home leaving for either marital or non-marital reasons), death of the research person, loss from observation, 23rd birthday, or January 1, 1940, when personal cards where introduced in the entire country. Moreover, research persons are obviously censored when their second biological parent dies which would automatically lead to the exit out of the parental household. Based on the historical context described above, in our empirical analyses we decided to study the leaving home behaviour for children aged 14 to 23. We consider children from age 14 onwards as working-age children which is in line with earlier research on the Netherlands (Bras & Kok, 2004). With regard to the upper age limit, children below the age of 23 were regarded as minors and had to be under the authority of an adult, typically a parent (van Solinge et al., 2000). Focussing on the leaving home behaviour of minor children allows us to thoroughly examine the power relations between the widowed parent and his/her minor children that were described in the background section. We additionally ran robustness checks with age 25 as the upper age limit, and the regression results remain robust to adjusting the upper age limit.

Children below marriage age (14–17) and children of marriage age (18–23) will be considered separately, and among the second group we will differentiate by the route out of the parental home. This will be done by running competing risks models which measure the relative risks of leaving home for marital or non-marital reasons (Fine & Gray, 1999). Using competing risks models with two or more reasons for leaving home is a frequently used approach in the demographic and sociological leaving home literature, and the dichotomy between leaving to live with a partner and for other reasons than partnership is most common (Blaauboer & Mulder, 2010; Buck & Scott, 1993; De Jong Gierveld et al., 1991; Holdsworth, 2000; Lundh & Öberg, 2018; Mulder & Clark, 2000). In this regard, Mulder and Clark (2000, p. 424) argue that “the use of a competing risks model established the reality of different processes of leaving home, a reality that emphasises the way in which differences in age, education, and not least, employment and income variations, play a role in creating the paths to adulthood.” This argument does not only apply to contemporary but also to historical populations, making competing risks models the appropriate choice for studying the leaving home behaviour of children of marriage age. Unfortunately, the data do not allow us to further separate the second group into leaving for work-related purposes on the one hand and for schooling and further education on the other. Nevertheless, the large majority of children that left for non-marital reasons left for work-related purposes as studying at boarding schools and universities was restricted to children (and especially sons) originating from the upper class and to those going to Catholic boarding schools. Male children that were drafted to serve in the army typically did not inform the municipality of residence about a change in address.

In all Cox regressions, we tested for the proportional-hazards assumption on the basis of Schoenfeld residuals. These tests indicate a non-violation of the proportional-hazards assumption of the main independent variable related to family structure. In some regressions, however, the control variables socioeconomic status, birth province, and living in an urban/rural area violate the proportional-hazards assumption. In these cases, we ran robustness checks in which we stratify for the respective control variables. These robustness checks indicate that the main results with regard to parental death are not sensitive to the inclusion of control variables that violate the proportional-hazards assumption.

### Variables

The main independent variable of interest is the child’s family structure which is introduced as a time-varying variable. In Sect. 4.1, which tests the first set of hypotheses, we introduce a time-varying dummy variable measuring whether a child experienced parental death or not. In Sect. 4.2, which tests the second set of hypotheses, we introduce two different time-varying categorical variables in two separate models. The first categorical variable differentiates between the sex of the deceased parent and the child’s age at parental death (0–5, 5–12, 12–18, 18–23). The second categorical variable controls for the presence of step-parents and the child’s age at which the step-parent entered the household (before or after age 12 in line with Hypotheses 2a and 2c).

In the regression analyses, we control for several other variables that are expected to be associated with the age at leaving home. First, earlier research has shown that parental social class is an important determinant for the transition to adulthood in general and the age at leaving home in particular, both among historical and contemporary populations (Blaauboer & Mulder, 2010; Bras & Kok, 2004; Furstenberg, 2008). We will use the father’s highest occupational status ever recorded on the civil certificates and the population registers as a proxy for the family’s socioeconomic status. With the help of a dataset provided by Mandemakers et al. (2013), the occupations are translated into the categorical classification scheme HISCLASS which groups occupations into twelve, seven, or five hierarchical social classes (van Leeuwen & Maas, 2011). Five social classes are used in this article in order to avoid small numbers in the specific categories. These classes are called elite, lower middle class (mainly white-collar workers), skilled workers, self-employed farmers and fishermen as well as unskilled workers.

In the period of consideration, religious denomination played a very important role in Dutch society and daily life. For instance, Catholic women typically married much later than their Liberal Protestant counterparts because they relied on marriage restraint as a traditional Malthusian method to limit family size (Engelen & Kok, 2002; Rosenbaum-Feldbrügge & Debiasi, 2019). In the analysis, religious denomination is divided into five categories: Catholic, liberal Protestant, orthodox Protestant, Jewish, and unknown/other. Members of the Dutch Reformed Church living in communities that solely elected orthodox ministers are regarded as orthodox Protestants (Kok, 2017). Household variables of interest are the number of older and younger sisters and brothers who survived until the age of 10, and the mother’s age at the research person’s birth. It is expected that the presence of many siblings generally reduces the age at leaving the parental home due to the high competition for resources within the household.

Finally, we also control for context characteristics. First, we differentiate between individuals born in the countryside and in cities. Municipalities are considered urban when they had more than 10,000 inhabitants and less than 2.5% of the population employed in the agricultural sector according to a nationwide census taken in 1899 (Kooij, 1985). Second, in order to cover cultural and economic differences within the country, the birth provinces are classified into four regions: North (Groningen, Friesland), West (North Holland, South Holland, Zeeland, Utrecht), South (North Brabant, Limburg), and East (Overijssel, Gelderland, Drenthe). To conclude, in line with earlier research we divide the period of observation in three birth periods (van Poppel et al., 2013): 1850–1879, 1880–1899, and 1900–1922. Bras and Kok (2004) found that the age of leaving home increased in the beginning of the twentieth century and explain this result with educational expansion, extended means of transportation, and the general decline in life-cycle service. The summary statistics of the variables by sex of the research person are depicted in Table 1. There are no considerable differences between male and female research person. Slightly more than 80% of the person-years were spent with both parents, and children were more likely to experience the entry of a step-mother than of a step-father. Only very few children were growing up with step-siblings.

**Table 1:** Summary statistics of the independent variables, by sex of the research person

	Women	Men
Family structure, %		
Both parents	79.8	78.6
Only father	5.6	5.5
Father and step-mother	3.6	4.6
Only mother	9.5	9.6
Mother and step-father	1.6	1.8
Stepsibling ever present, %		
No	98.9	98.5
Yes	1.1	1.5
Highest paternal HISCLASS, %		
Elite	4.3	3.6
Lower middle class	22.7	21.8
Skilled worker	35.3	34.7
Self-employed farmer and fisherman	16.9	17.3
Unskilled worker	20.7	22.6
Religion, %		
Catholic	32.8	32.8
Liberal Protestant	42.1	42.0
Orthodox Protestant	15.3	15.5
Jewish	1.5	1.6
Unknown/other	8.3	8.1
Total number siblings	5.1	5.2
Mother’s age at birth, %		
< 25	13.5	14.0
25–35	58.6	60.3
> 35	27.9	25.7
Period of birth, %		
1850–1879	26.2	25.9
1880–1899	37.6	37.4
1900–1922	36.2	36.7
Birth region, %		
North	15.1	14.8
West	52.9	53.0
East	17.3	17.4
South	14.8	14.8
Birth municipality, %		
Rural	65.6	66.4
Urban	34.4	33.6
Number of individuals	10,655	10,788
Number of events	4490	2998
Person years	76,808	84,089

## Results

### The Determinants of the Routes of Leaving Home

**Fig. 1:**
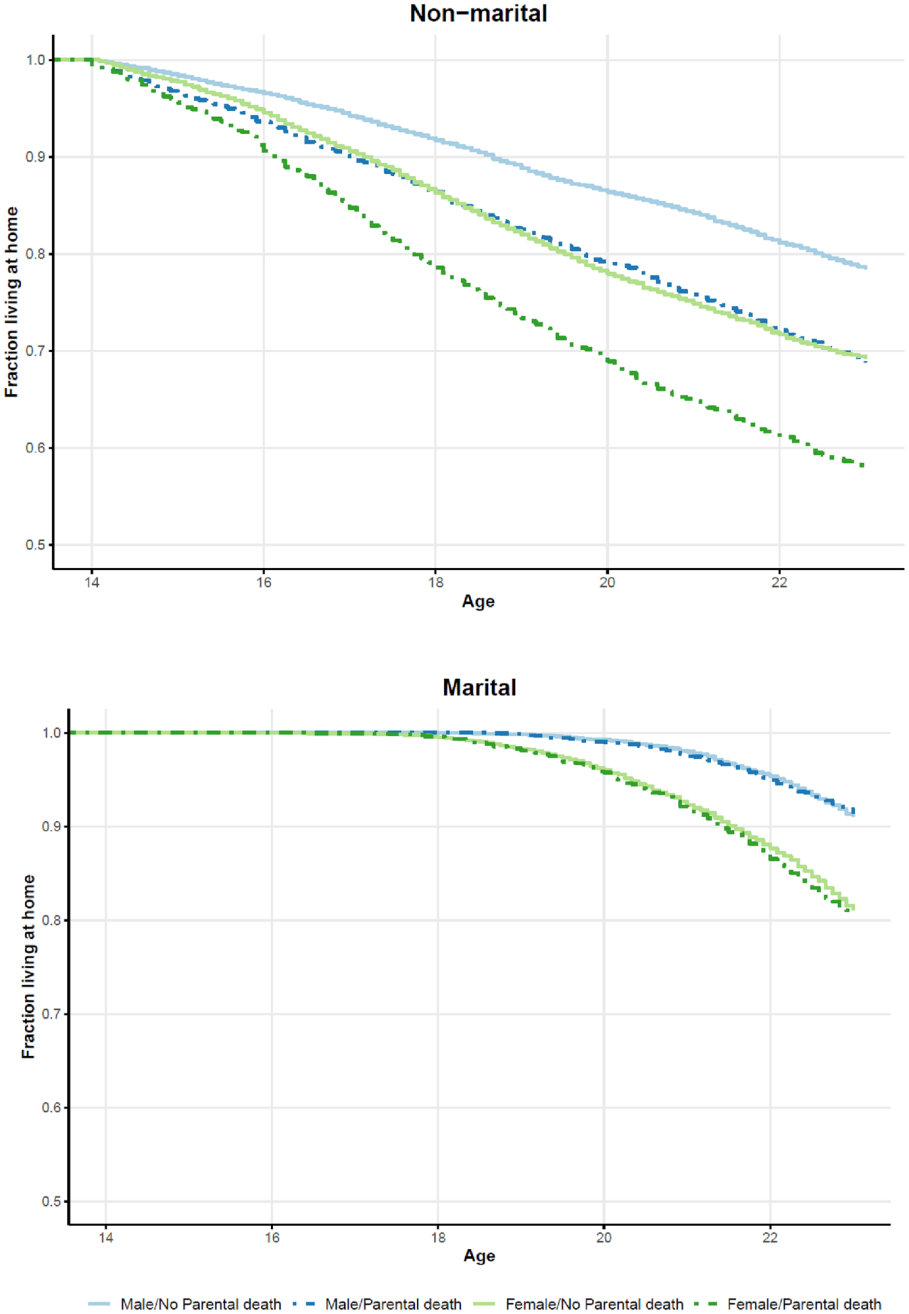
Kaplan–Meier curves for leaving the parental home for non-marital and marital reasons, by sex of the research person and having experienced parental death

In this section, we closely examine the determinants of leaving home for marital and non-marital reasons. Figure 1 shows Kaplan–Meier curves representing the male (blue) and female (green) individuals who left either for non-marital (upper graph) or marital (lower graph) reasons between the ages 14 and 23. The dashed lines represent children that lost a parent, and the solid lines represent those who did not experience parental death. As in the event-history analysis presented below, parental death is treated as a time-varying dummy variable. The upper graph shows that the first children left the parental home for non-marital reasons at around age 14 and that both half-orphaned daughters and sons left home earlier than their non-orphaned counterparts. This is in line with Hypothesis 1a. The lower graph reveals that Dutch children in the period of consideration basically did not marry before age 18. Furthermore, the graph indicates that the marriage behaviour of half-orphaned and non-orphaned children did not differ considerably, which supports Hypothesis 1b. In addition, the graphs in Fig. 1 also show that daughters generally moved out of the parental household earlier than sons, which is also found in research on contemporary western populations (Billari et al., 2001). Due to these gender differences in ages at leaving home, it is reasonable to run separate regressions for daughters and sons.

Table 2 depicts the regression results for female and Table 3 the results for male research persons. Model 1 in both tables studies children below the age of 18 and only analyses leaving home for non-marital reasons as marriage basically did not occur before the age of 18. In Model 2 and Model 3, we study the age range 18–23 and introduce competing risk models with leaving home for non-marital and marital reasons as competing outcomes. In both tables, parental death is introduced as a time-varying dummy variable.

**Table 2:** Event-history analysis for first leaving home for marital and non-marital reasons, women

	(1)		(2)		(3)	
	Non-marital living		Non-marital living		Marriage	
	Ages 14–18		Ages 18–23		Ages 18–23	
	Hazard ratio	95% CI	Hazard ratio	95% CI	Hazard ratio	95% CI
Parental death						
Yes	1.59***	1.42–1.79	1.38***	1.23–1.54	1.03	0.90–1.18
No	Ref	Ref	Ref	Ref	Ref	Ref
Father’s highest HISCLASS						
Elite	0.79	0.59–1.06	1.06	0.83–1.35	0.53***	0.37–0.77
Lower middle class	0.93	0.80–1.07	0.99	0.87–1.13	0.82*	0.70–0.96
Skilled worker	Ref	Ref	Ref	Ref	Ref	Ref
Farmer or fishermen	0.76**	0.65–0.90	0.70***	0.60–0.83	0.82*	0.68–1.00
Unskilled worker	1.27***	1.11–1.44	1.26***	1.11–1.43	1.44***	1.24–1.66
Religion						
Liberal protestant	Ref	Ref	Ref	Ref	Ref	Ref
Catholic	1.09	0.95–1.25	1.03	0.90–1.17	0.70***	0.60–0.82
Orthodox protestant	0.90	0.77–1.05	0.93	0.80–1.08	0.79**	0.66–0.94
Jewish	0.40*	0.19–0.85	0.93	0.62–1.39	0.70	0.43–1.13
Unknown/other	0.94	0.77–1.14	1.00	0.83–1.21	1.08	0.88–1.31
Number older brothers	0.98	0.94–1.03	1.01	0.97–1.05	1.09***	1.04–1.13
Number older sisters	1.09***	1.04–1.13	1.04	1.00–1.08	1.03	0.99–1.08
Number younger brothers	1.05*	1.00–1.09	1.04*	1.00–1.08	0.99	0.95–1.04
Number younger sisters	1.16***	1.12–1.20	1.12***	1.08–1.16	1.03	0.99–1.07
Period of birth						
1850–1879	Ref	Ref	Ref	Ref	Ref	Ref
1880–1899	1.01	0.90–1.15	1.10	0.98–1.24	1.05	0.91–1.21
1900–1922	0.77***	0.67–0.88	0.74***	0.65–0.85	0.98	0.84–1.14
Mother’s age at birth						
< 25	0.89	0.76–1.04	0.90	0.77–1.05	1.70***	1.45–1.98
25–35	Ref	Ref	Ref	Ref	Ref	
> 35	0.94	0.82–1.09	0.99	0.86–1.13	0.88	0.75–1.04
Municipality of birth						
Urban	0.64***	0.57–0.73	1.02	0.91–1.15	1.21**	1.06–1.38
Rural	Ref	Ref	Ref	Ref	Ref	Ref
Region of birth						
West						
North	1.52***	1.32–1.74	1.39***	1.21–1.60	0.66***	0.55–0.79
East	1.07	0.93–1.23	1.20**	1.05–1.37	0.61***	0.51–0.73
South	0.93	0.78–1.11	0.77**	0.64–0.92	1.06	0.87–1.28
Number of subjects	10,655		8599		8599	
Number of failures	1569		1627		1251	

*p < 0.05, **p < 0.01, ***p < 0.001

**Table 3:** Event-history analysis for first leaving home for marital and non-marital reasons, men

	(1)		(2)		(3)	
	Non-marital living		Non-marital living		Marriage	
	Ages 14–18		Ages 18–23		Ages 18–23	
	Hazard ratio	95% CI	Hazard ratio	95% CI	Hazard ratio	95% CI
Parental death						
Yes	1.55***	1.34–1.79	1.45***	1.28–1.63	0.94	0.79–1.13
No	Ref	Ref	Ref	Ref	Ref	Ref
Father’s highest HISCLASS						
Elite	2.12***	1.59–2.84	1.55***	1.21–1.99	0.23***	0.10–0.52
Lower middle class	1.33**	1.11–1.60	1.04	0.90–1.19	0.90	0.74–1.11
Skilled worker	Ref	Ref	Ref	Ref	Ref	Ref
Farmer or fishermen	1.34**	1.11–1.62	0.65***	0.54–0.78	0.45***	0.33–0.62
Unskilled worker	1.29**	1.08–1.54	0.86	0.75–1.00	1.35**	1.12–1.63
Religion						
Liberal protestant	Ref	Ref	Ref	Ref	Ref	Ref
Catholic	1.15	0.97–1.37	0.88	0.75–1.02	0.71**	0.57–0.88
Orthodox protestant	0.84	0.68–1.02	0.91	0.77–1.08	1.04	0.83–1.29
Jewish	0.73	0.37–1.42	1.32	0.92–1.90	1.00	0.60–1.68
Unknown/other	0.98	0.77–1.26	0.88	0.71–1.09	0.77	0.56–1.06
Number older brothers	1.04	0.99–1.09	1.03	0.99–1.07	1.12***	1.05–1.19
Number older sisters	0.97	0.92–1.02	1.01	0.96–1.05	1.05	0.98–1.11
Number younger brothers	1.05*	1.00–1.10	0.99	0.95–1.03	1.04	0.98–1.10
Number younger sisters	1.03	0.98–1.08	0.98	0.94–1.02	1.01	0.95–1.07
Period of birth						
1850–1879	Ref	Ref	Ref	Ref	Ref	Ref
1880–1899	1.05	0.90–1.22	1.08	0.94–1.23	0.89	0.73–1.08
1900–1922	0.76**	0.64–0.89	0.97	0.84–1.12	0.88	0.72–1.07
Mother’s age at birth						
< 25	0.85	0.68–1.05	0.96	0.81–1.15	1.71***	1.38–2.11
25–35	Ref	Ref	Ref	Ref	Ref	Ref
> 35	1.12	0.94–1.33	0.97	0.83–1.13	0.87	0.68–1.10
Municipality of birth						
Urban	0.74***	0.63–0.87	0.93	0.82–1.06	1.38***	1.16–1.64
Rural	Ref	Ref	Ref	Ref	Ref	Ref
Region of birth						
West	Ref	Ref	Ref	Ref	Ref	Ref
North	1.94***	1.63–2.30	1.43***	1.23–1.66	0.84	0.66–1.06
East	1.32**	1.10–1.57	1.18*	1.01–1.37	0.75*	0.59–0.96
South	0.97	0.78–1.21	0.86	0.70–1.06	0.85	0.63–1.14
Number of subjects	10,788		9396		9396	
Number of failures	977		1355		665	

*p < 0.05, **p < 0.01, ***p < 0.001

In accordance with Hypothesis 1a and Fig. 1, Model 1 shows that parental loss during childhood and adolescence was significantly associated with earlier home leaving among sons and daughters below age 18. The effect sizes are 1.59 for daughters and 1.55 for sons. The age at leaving home for non-marital reasons is also reduced by parental death for children aged 18 to 23, as shown in Model 2. Again, the effect sizes for daughters and sons are similar, but a little bit lower compared to Model 1. Model 3 supports Hypothesis 1b because parental death among daughters and sons was not associated with leaving home for early marriage. Consequently, there is strong evidence for Hypotheses 1a and 1b which state that experiencing parental death was associated with earlier home leaving for non-marital reasons, but did not increase the chance for early marriage.

With regard to the remaining covariates, the results show that social class was a very important determinant for children’s leaving home behaviour and that the effect of social class varied by the sex of the child and the route out of the parental home. First of all, children of the elite were least likely to enter into an early marriage. Family controls on partner selection were strongest among this group to prevent early marriages which were considered socially inappropriate (Janssens, 2014). As family controls were less strong among the lowest social class and also material thresholds of starting a household were lower, children of unskilled workers were most likely to enter into an early marriage. Children of unskilled workers, and daughters in particular, were also most likely to leave the parental household for non-marital reasons which points to the importance of life-cycle service within the life course of lower skilled individuals. Daughters of farmers were most likely to stay with their parents during adolescence, whereas farmer’s sons somewhat surprisingly also participated in life-cycle service and probably worked at other farms to gain experience. Remarkably, elite sons had the highest risk of leaving the parental home for non-marital reasons compared to all other social groups. This is explained by the fact that the upper-classes often sent their male children to boarding schools and universities (Janssens, 1993).

Catholic children and also orthodox protestant girls were much less likely to marry before the age of 23 because early marriages were not in the line with the social norms dominant within these groups (Engelen & Kok, 2002). Jewish daughters were least likely to leave their parents before the age of 18. The number of siblings generally increased daughters’ risks of leaving home, especially for non-marital living and for those who had many older and younger sisters. Sons’ leaving home behaviour was much less affected by the sibship size. Mother’s age at birth did not have an impact on the age at leaving home for non-marital living, but having a young mother strongly increased the chances for early marriage, both for male and female children. This indicates that marriage behaviour was to a certain degree transmitted between generations and is in line with earlier findings (van Bavel & Kok, 2009).

With regard to the context characteristics, the propensity of early marriage did not change over time, neither for men nor for women. But being born between 1900 and 1922 was associated with a lower chance for leaving for non-marital reasons. This points to the decline in life-cycle service in the beginning of the twentieth century (Bras & Kok, 2004). Living in an urban area had opposing effects on leaving home behaviour than living in a rural area. Boys and girls born in rural areas were significantly more likely to leave the parental home below age 18 than children born in cities. On the contrary, being born in an urban area conveys higher hazard ratios for early entry into marriage. Finally, also the region of residence mattered with regard to leaving home. Particularly daughters born in the Northern and Eastern parts were less likely to enter marriage before the age of 23. With regard to leaving for non-marital reasons, however, the opposite pattern was observed because children born in the North of the country were most likely to leave for work.

### The Impact of the Family Structure on Age at Leaving Home

In Tables 4 and 5, we study the role of the family structure in more detail for daughters and sons, respectively. In this section, we analyse the entire age group 14–23 and only study leaving for non-marital reasons as it has been shown before that leaving for marriage is not affected by the loss of a parent. Furthermore, parental death is considered as a time-varying categorical variable. In Model 1, we differentiate between maternal and paternal death and the age at which the child experienced parental death. In Model 2, we take the presence of step-siblings and of step-parents into account who entered before and after the age of 12. In Tables 4 and 5, we control for the same covariates as above, but they are not shown in the output tables because they convey basically the identical information as the first two models in Tables 2 and 3.

**Table 4:** Event-history analysis for the impact of family structure on first leaving home for non-marital reasons, women

	(1)		(2)	
	Age and sex		Presence of step-parents	
	Ages 14–23		Ages 14–23	
	Hazard ratio	95% CI	Hazard ratio	95% CI
Age at parental death and sex of deceased parent				
No parental death	Ref	Ref		
Maternal death, 0–5	1.68***	1.36–2.07		
Paternal death, 0–5	1.12	0.90–1.41		
Maternal death, 5–12	1.66***	1.42–1.94		
Paternal death, 5–12	1.38***	1.18–1.63		
Maternal death, 12–18	1.62***	1.36–1.94		
Paternal death, 12–18	1.41***	1.19–1.67		
Maternal death, 18–23	1.62**	1.17–2.26		
Paternal death, 18–23	1.33	0.95–1.86		
Presence of step-parents				
No parental death			Ref	Ref
Maternal death			1.62***	1.42–1.86
Maternal death, Stepmother < 12			1.77***	1.48–2.11
Maternal death, Stepmother ≥ 12			1.34*	1.01–1.77
Paternal death			1.30***	1.15–1.46
Paternal death, Stepfather < 12			1.14	0.85–1.54
Paternal death, Stepfather ≥ 12			2.17***	1.51−3.12
Presence of step-siblings				
No			Ref	Ref
Yes			1.24	0.94–1.63
Individuals	10,655		10,655	
Failures	3196		3196	

*p < 0.05, **p < 0.01, ***p < 0.001

**Table 5:** Event-history analysis for the impact of family structure on first leaving home for non-marital reasons, men

	(1)		(2)	
	Age and sex		Presence of step-parents	
	Ages 14–23		Ages 14–23	
	Hazard ratio	95% CI	Hazard ratio	95% CI
Age at parental death and sex of deceased parent				
No parental death	Ref	Ref		
Maternal death, 0–5	1.34*	1.04–1.73		
Paternal death, 0–5	0.84	0.63–1.13		
Maternal death, 5–12	1.63***	1.37–1.94		
Paternal death, 5–12	1.33**	1.10–1.60		
Maternal death, 12–18	1.93***	1.59–2.32		
Paternal death, 12–18	1.47***	1.22–1.79		
Maternal death, 18–23	2.60***	1.92–3.51		
Paternal death, 18–23	1.37	0.98–1.92		
Presence of step-parents				
No parental death			Ref	Ref
Maternal death			1.81***	1.56–2.09
Maternal death, Stepmother < 12			1.59***	1.29–1.96
Maternal death, Stepmother ≥ 12			1.84***	1.42–2.38
Paternal death			1.31***	1.15–1.50
Paternal death, Stepfather < 12			0.96	0.65–1.42
Paternal death, Stepfather ≥ 12			1.36	0.83–2.20
Presence of step-siblings				
No			Ref	Ref
Yes			0.91	0.66–1.26
Individuals	10,788		10,788	
Failures	2332		2332	

*p < 0.05, **p < 0.01, ***p < 0.001

Model 1 in Tables 4 and 5 reveals that maternal death in all age groups is significantly associated with earlier home leaving among both boys and girls aged 14 to 23. Paternal death, in contrast, is only significantly associated with earlier home leaving among children aged 5–18, implying that boys and girls experiencing a father’s death in early childhood (0–5) and young adulthood (18–23) do not leave the parental home earlier than their non-bereaved counterparts. For female children aged 0–5 and for male children aged 0–5, 12–18 and 18–23 joint tests reveal that the differences between maternal and paternal death are also statistically significant, meaning that these children are more likely to leave the parental household when losing a mother than a father. With respect to differences by children’s age at parental death, boys who lost a mother before age 5 reported significantly lower hazard ratios than those who experienced maternal death aged 12–18 and aged 18–23. This implies that boys who lost a mother very early in life were less likely to leave the paternal household for non-marital reasons than those who experienced maternal death in adolescence and early adulthood. Among girls, in contrast, maternal death across all age groups is to a very similar degree associated with early home leaving.

Model 2 indicates that children who shared a household with step-parents and step-siblings generally showed a very similar leaving home behaviour compared to children whose widowed parent had not remarried yet. According to the joint tests performed, only daughters left the parental household earlier who lived together with a step-father who entered after their twelfth birthday, compared to living with a single widowed mother (*F* = 7.21, *p* > *F* = 0.01) and compared to having a step-father who entered before the age of 12 (*F* = 7.49, *p* > *F* = 0.01). Apparently, in most cases it was not the entry of a step-parent and step-siblings that made the children leave their parental home, but the death of a parent as such. Taken together, this does not support Hypotheses 2a, 2b, and 2c.

## Concluding Discussion

In this article, we analysed the leaving home behaviour of more than 21,000 minor children born in the Netherlands in the period 1850–1922. As described in the results section, the covariates were mostly in line with earlier research on the Netherlands and showed that especially the socioeconomic and religious background had a strong impact on leaving home in the period of consideration (Bras & Kok, 2004; Engelen & Kok, 2002). Furthermore, urban children displayed a very different leaving home behaviour than rural children as they were less likely to leave the parental home below age 18 for non-marital reasons, but more likely to enter an early marriage.

In line with research on contemporary western societies, this article shows that the availability of pathways out of the parental home also plays an important role when studying leaving home behaviour in the past. In the period of consideration, the majority of children had fewer opportunities to leave the parental household compared to children growing up in contemporary Western society. Premarital cohabitation was not in accordance with the social norms at that time and only privileged children had the chance to leave for educational purposes. All other children basically only had the choice between leaving home for work-related reasons or, once they had reached age 18, marriage. The access to marriage, however, was strongly restricted by the prevailing religious norms and financial constraints. Religious denominations such as the Catholic Church and orthodox Protestants as well as higher social classes disapproved of early marriages, and children additionally had to accumulate enough savings to establish their own nuclear household upon marriage. Beyond that, parents had a crucial influence on their offspring’s marital decisions as they were entitled to object to the marriages of their adult children up until age 30. Therefore, leaving the parental household for marriage was for many children not a reasonable option in early adulthood. As a consequence, also children aged 18 to 23 predominantly left the parental home for non-marital reasons. The results of the article therefore show that the opportunity structures directed by society’s norms and constraints strongly shaped the leaving home behaviour of adolescent and adult children. These opportunity structures additionally varied between rural and urban areas, thereby indicating that children’s leaving home opportunities were also directed by local labour and marriage markets.

The findings of the article also suggest that the effect of parental death differed by the pathway out of the parental home. First, the results indicate that the marriage behaviour in young adulthood was unaffected by parental death. As discussed before, this might be explained by the strict constraints imposed on marriage in the period of consideration. Early parental death often resulted in the family’s lower standard of living (Oris & Ochiai, 2002), which in turn complicated an early and smooth transition to marriage (Rosenbaum-Feldbrügge & Debiasi, 2019). Moreover, widowed parents probably did not support the early marriage of their offspring because married children did not contribute to the household budget any longer and were lost as productive assets (de Regt, 2004). Accordingly, it is not surprising that children did not hasten into marriage after having experienced the untimely death of a parent.

Second, and in line with the expectations, the loss of a parent was associated with a much higher chance of leaving the parental household for non-marital reasons. Remarkably, maternal death frequently resulted in higher risks for leaving home than paternal death. In contrast to our expectations, the entry of a step-parent and step-siblings was generally not associated with a higher likelihood of leaving the parental home compared to children who were living with a single widowed parent. Only the entry of step-fathers after the twelfth birthday was associated with an increased chance of home leaving for daughters. Our results therefore point to the direction that tensions caused by the entry of a step-parent and step-siblings in the household do not fully explain parentally bereaved children’s higher likelihood of leaving the parental household. This finding is at odds with suggestions provided by earlier research (Bras & Kok, 2004; Dribe, 2000).

If it is not tensions with the step-parents, what else could cause the earlier home leaving of parentally bereaved children? As described earlier, leaving home for non-marital reasons was subject to a joint decision made by parents and their offspring and therefore based on mutual interests (Dribe, 2000). In the following, we therefore argue that both the child and the widowed parent had an interest in the offspring’s earlier home leaving. From the parental perspective, the exit of the working-age child can firstly be seen as a budgetary relief because parents saved expenses on food and clothing (Bras & Kok, 2004). As unmarried children living outside the parental home often contributed to the family budget, parents had an increased interest in sending their working-age children to employers to save money and still generate some income. Second, life-cycle servants were typically hired for an entire year. Sending children away to work as a life-cycle servant therefore minimized the short-term risk of unemployment and ensured a steady source of income (Paping, 1999). In contemporary developing countries where insurance markets and welfare states are underdeveloped, work migration of adolescent and adult children is often seen as a way to diversify risks (Massey et al., 1993). A similar phenomenon is possibly observed in the Netherlands in the period of consideration where widowed parents had a strong interest in their children finding stable jobs in different economic sectors to be able to support the family financially even in times of economic hardship.

From the child’s perspective, the loss of a parent was first and foremost a disrupting event that caused economic strains and most probably resulted in decreasing living standards. Possibly, bereaved families without property were also forced to move to a smaller dwelling as residential moves were a typical coping strategy frequently applied by the urban poor (Kok et al., 2005). A drop in living standards, in turn, made staying at the parental home less attractive and increased the children’s incentive to leave to provide to a certain extent for themselves. The results also indicate that living with a widowed father was less attractive than living with a widowed mother as maternal death was associated with a very high risk of leaving the parental home. This finding is in line with earlier research showing that widowed mothers were better able to prevent the dissolution of the family unit than widowed fathers (Humphries, 2010; Rosenbaum-Feldbrügge, 2018).

With regard to limitations, just as Bras and Kok (2004) we want to emphasize the potential shortcomings of the population register as a source for studying children’s leaving home behaviour for non-marital reasons. Even though the registration of migratory moves to another municipality was compulsory in the study period, quite a few persons failed to comply with the registration of their migration. In that case, their absence was noted with reference to the date of the census conducted every ten years. Accordingly, the exact timing of home leaving may be registered incorrectly on the population register to some extent. This limitation, however, does not apply to home-leaving for marital reasons as the date of marriage available on the marriage certificate usually corresponds with the date of leaving home as stated on the population register.

In conclusion, the present article showed that children’s leaving home behaviour in the Netherlands in the nineteenth and beginning of the twentieth century was determined by socioeconomic constraints, religious norms, geographic contexts, and changes in the household composition. While we are confident that our results are generalizable for other areas characterized by the Western European marriage pattern in the past (see also Lundh & Öberg, 2018), future research could extend the scope of our analysis by studying the determinants of home leaving in other regions frequently studied by historical demographers such as Southern and Eastern Europe as well as East Asia. Finally, research on leaving the parental home in the Netherlands so far is divided into the period before and after World War Second (see for instance Blaauboer & Mulder, 2010 for leaving home in the second half of the twentieth century). Combining both periods in one analysis that covers more than 150 years would certainly enhance our knowledge about long-term trends in leaving home in Western Europe.

## Data Availability

By means of the HSN download service, one can download public and non-public HSN data after getting confirmation by email from HSN.

## Code Availability

R and STATA code is available upon request.
